# The Association between Serum Leptin and Post Stroke Depression: Results from a Cohort Study

**DOI:** 10.1371/journal.pone.0103137

**Published:** 2014-07-25

**Authors:** Yan-tao Li, Ye Zhao, Hua-jing Zhang, Wen-li Zhao

**Affiliations:** 1 Department of Prevention and Health Care, Tianjin Nankai Hospital, Tianjin, P. R. China; 2 Department of Clinical Research, Tianjin Nankai Hospital, Tianjin, P. R. China; 3 Department of Pediatrics, Tianjin Wuqing Hospital of Traditional Chinese Medicine, Tianjin, P. R. China; 4 Department of Neurology, Tianjin Nankai Hospital, Tianjin, P.R. China; Institute of Psychiatry, United Kingdom

## Abstract

**Background:**

Depression is a frequent mood disorder that affects around a third of stroke patients and has been associated with poorer outcomes. Our aim was to determine whether there was a relationship between inflammatory markers (leptin) and post-stroke depression (PSD).

**Methods:**

One hundred and ninety-one ischemic stroke patients admitted to the hospital within the first 24 hours after stroke onset were consecutively recruited and followed up for 3 months. Enzyme-linked immunosorbent assay (ELISA) was used to measure serum levels of leptin at admission. Based on the symptoms, diagnoses of depression were made in accordance with DSM-IV criteria for post-stroke depression at 3 month.

**Results:**

Forty-four patients (23.0%) were diagnosed as having major depression at 3 month. Patients with depression showed higher serum leptin levels at 3 month after stroke (32.2 [IQR, 20.8–57.7] v. 9.9 [IQR, 4.6–13.1]ng/ml, respectively; *P* = 0.000). Serum levels of leptin ≥20 ng/ml were independently associated with PSD [odds ratio (OR) 20.23, 95% confidence interval (CI) 9.11–51.26, *P* =  0.000], after adjusting for possible confounders.

**Conclusions:**

Serum leptin levels elevated at admission were found to be associated with PSD and may provide a new proposal for the treatment of PSD.

## Introduction

Depression is particularly prevalent among stroke survivors, affecting approximately a third of individuals [1]. China has 2.5 million new stroke cases each year and 7.5 million stroke survivors [2]. Thus, there will be 3.0 million stroke patients with depression. Patients with depression experience worse stroke-related outcomes in the form of greater functional disability and higher mortality [3], and, finally with worse rehabilitation outcomes [4]. Moreover, an improvement of depressive symptoms has been associated with a better functional recovery [4]. Early recognition of depression symptoms and introduction of pharmacological treatment is of great importance in the reduction of stroke complications and stroke mortality as well as for better functional outcomes [5], making the prevention and management of post-stroke depression (PSD) an important area of research.

Chronic inflammation has been suggested as an important mechanism related to depression [6]–[7], such as: C-reactive protein (CRP), interleukin-6 (IL-6), IL-1 [8], IL-18[9]. Likewise, Kim et al [10] reported that reduced anti-inflammatory cytokine function was associated with PSD, supporting the cytokine hypothesis in its etiology.

Leptin is a hormone secreted by adipose tissue in direct proportion to amount of body fat. It circulates as a 16-kDa protein and is transported across the blood–brain barrier by a saturable system to exert its central effects [11]. Its main function are focused on behavior and energy balance control [12]. In addition, it has been studied on account of its participation in anti-obesity [13], neuroprotection[14], reducing oxidative stress[15], protective and harmful effects on the cardiovascular (CV) system[16] and as a vascular risk factor for stroke[17].

Lu et al [18] reported that leptin has been associated with the development of depression in clinical and experimental studies. Whereas, some authors have found higher leptin levels in patients with depression [19]–[20], others have reported lower leptin levels [21]–[22]. Thus, the role of leptin in patients with depression remains inconsistent. Interestingly, there was rare study on serum leptin levels in patients with PSD. Therefore, our aim was to determine whether there was a relationship between leptin and PSD in Chinese population.

## Methods

### Study population

Two hundreds and fifty-six patients with a first episode of acute ischemic stroke admitted to our hospital within the first 24 h of stroke onset were prospectively included in the study. Patients were included if they were admitted to the emergency department with an acute ischemic stroke defined according to the World Health Organization criteria [23]. Patients with subarachnoid or intracranial hemorrhage, decreased level of consciousness, severe aphasia or dysarthria, or psychiatric illness, severe infectious or inflammatory diseases, and life expectancy <3month were excluded.

One hundred and forty-five out of 256 patients (56.6%) were male, with a mean age of 68.6±10.1 years. Sixty-five patients were not evaluated at 3 month (thirty-one patients died and 9 refused to attend the follow-up, nine patients had difficulty in being transported to hospital, and 16 patients were lost to follow-up); the remaining 191 patients were valid for analysis. Written informed consent was obtained after having provided verbal and written information to participants or nearest relatives when relevant. Ethics approval was granted by The Ethics Committee for Medical Research at Tianjin Nankai Hospital.

### Clinical variables

At baseline, age, sex, body mass index and history of risk factors were obtained. Stroke subtype was classified according to TOAST (Trial of ORG 10172 in Acute Stroke Treatment) criteria [24]. Routine blood and biochemical tests, brain CT/MRI scan were performed in all patients at admission. MRI with diffusion-weighted imaging (DWI) was available in some patients. The infarct volume was calculated by using the formula 0.5×a×b×c (where a is the maximal longitudinal diameter, b is the maximal transverse diameter perpendicular to a and c is the number of 10-mm slices containing infarct) [25].

Stroke severity was evaluated by trained neurologists using the NIHSS at admission [26]. Functional outcomes were evaluated by the modified Rankin Scale (mRS) at 3 month [27]. A favorable functional outcome was defined as a mRS score of 0 to 2 points, while an unfavorable functional outcome was defined as a mRS score of 3 to 6 points.

### Psychological Measurement

Depression assessments were conducted by a neurologist/psychiatrist who was unaware of the type, size and location of the index stroke at the time of 3 months after stroke onset. Previous history of psychiatric disease and depression, educational level and people living with the patient were recorded at admission. Clinical depression was diagnosed according to DSM-III-R criteria [28] using algorithms based on psychiatric interview and neuropsychiatric examination. The presence of anhedonia and depressive mood was essential for the diagnosis.

### Laboratory tests

Fasting venous blood was collected from all participants in vacutainer tubes and quickly centrifuged to avoid glycolysis. Serum samples were kept at −80°C until assay. Biomarker concentrations were measured in a central laboratory by investigators blinded to the clinical outcomes and neuroimaging findings. The serum levels levels were measured using measured with a commercially available quantitative enzyme-linked immunosorbent assay (ELISA) kit (Abcam, China). The lower detection limit was 2 ng/ml and the line range was 2–400 ng/ml. The intra-assay coefficient of variation [CV] and inter-assay CV were 4.6–7.5% and 5.3%–8.8%, respectively.

### Statistical analyses

The results are expressed as percentages for categorical variables and as mean (standard deviation, S.D.) or median (interquartile range, IQR) for the continuous variables depending on their normal distribution. Correlations among continuous variables were assessed by the spearman rank-correlation coefficient. Proportions were compared using the Chi-square test, and the student's t test or the Mann–Whitney test was used to compare continuous variables between groups as appropriate. The influence of serum leptin levels on PSD was performed by binary logistic regression analysis, which allows adjustment for confounding factors (age, sex, body mass index(BMI), stroke syndrome, stroke etiology, the NIHSS score, infarct volume, vascular risk factors, others blood biomarkers and a history of depression). The results are expressed as adjusted odds ratios (ORs) with the corresponding 95% confidence intervals (CIs). Receiver operating characteristic (ROC) curves were utilized to evaluate the accuracy of serum leptin to predict PSD. Area under the curve (AUC) was calculated as measurements of the accuracy of the test. All statistical analysis was performed with SPSS for Windows, version 19.0 (SPSS Inc., Chicago, IL, USA). Statistical significance was defined as p<0.05.

## Results

The study cohort consisted of 256 patients at baseline (stroke admission). By the time of follow-up at 3 month, leaving 191 individuals were included in our study. The mean age was 68.5 (SD: 10.4) years and 44.0% were women. The median serum leptin on admission was 12.9[IQR, 6.6—20.8]ng/ml. The median NIHSS score on admission was 6 points (IQR, 3 to 11). At 3 month, seventy-nine patients (41.4%) showed depression and in 44 patients (23.0%) this depression was classified as major. The clinical variables associated with the presence of major depression at 3 month were shown in Table 1.

**Table 1:** Basal characteristic of patients with acute ischemic stroke

Baseline Characteristics	Depression patients (*n* = 44)	No depression (*n* = 147)	*P* a
Age (years), mean(SD)	71.2 (9.0)	64.9 (12.1)	0.009
Female sex, %	59.1	39.5	0.021
BMI(kg m^-2^, IQR)	26.8(23.1–29.4)	23.9(21.8–27.2)	0.001
Hypertension, %	47.7	49.7	0.822
Diabetes at baseline, %	40.9	37.4	0.693
Hypercholesterolemia, %	38.6	39.5	0.873
Coronary heart disease, %	29.5	27.2	0.717
Family history of stroke, %	22.7	20.4	0.401
Admission median NIHSS score (IQR)	7 (4–12)	6(3–10)	0.126
mRS at 3 month, median (IQR)	3(1–3)	2(1–3)	0.221
Infarct volume (ml), mean(SD)	10.8(1.4)	10.5(1.3)	0.671
Time from onset to inclusion (hr, IQR)	4.8 (2.3–11.2)	5.0(2.4–11.3)	0.452
Widowhood (%)	40.9	18.4	0.002
Living with offspring (%)	36.4	13.6	0.001
Days of hospitalization, median (IQR)	10(7–13)	9(5–12)	0.526
TOAST classification (%)			0.231
a. Large artery	15.9	17.0	–
b. Small artery	18.2	18.4	–
c. Cardioembolism	36.4	37.4	–
d. Other cause	13.6	14.3	–
e. Unknown	15.9	12.9	–
Laboratory findings			
White cell count, ×109/L (Median, IQR)	7.7 (5.9–8.6)	7.6 (5.5–8.4)	0.826
Glucose level, mmol/L (Median, IQR)	5.44(4.75–6.51)	5.38 (4.85–6.55)	0.324
Hs- CRP, mg/dL (Median, IQR)	0.88 (0.36–2.44)	0.40 (0.21–1.20)	0.003
HCY, umol/L (Median, IQR)	18.8(13.9–23.7)	14.6 (11.4–18.5)	0.023
Leptin (ng/ml)	32.2(20.8–57.7)	9.9(4.6–13.1)	<0.001

a Mann–Whitney U test, student's t test or Chi-square test was used.

Results are expressed as percentages or as medians (IQR) and means (SD); hs-CRP: high-sensitivity C-reactive protein; HCY: homocysteine; BMI, Body Mass Index.

**Figure 1:**
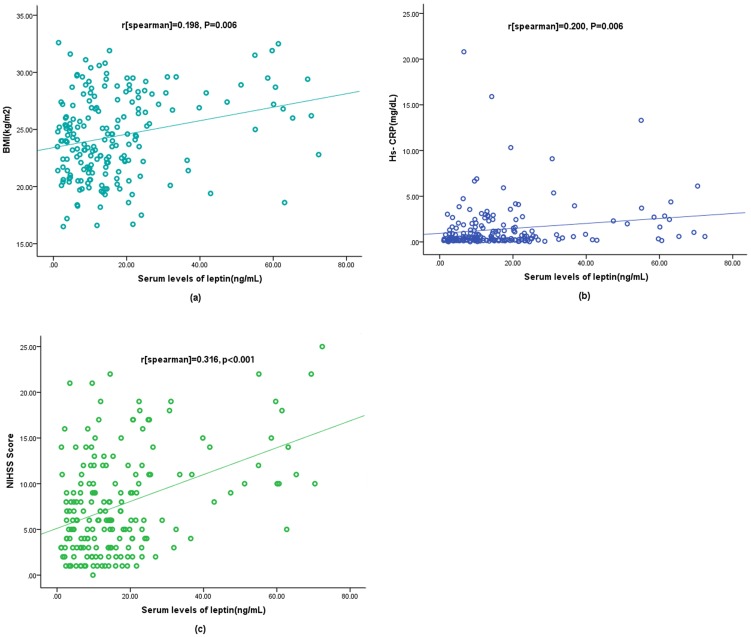
Correlation between serum leptin levels and others predictors. (a) Correlation between the serum leptin levels and BMI; (b) Correlation between serum leptin levels and Hs-CRP; (c) Correlation between the serum leptin levels and the National Institutes of Health Stroke Scale (NIHSS) score.

There was a positive correlation between serum levels of Leptin and BMI (*r* [spearman]  = 0.198, *P* = 0.006; Figure 1a). There was also a modest correlation between levels of serum Leptin levels and hs-CRP (*r* [spearman]  = 0.200, *P* = 0.006; Figure 1b). Leptin levels increased with increasing severity of stroke as defined by the NIHSS score. There was a modest correlation between serum levels of Leptin and NIHSS score (*r* [spearman]  = 0.317, *P*<0.001; Figure 1c). There were also slightly correlations between levels of serum leptin and sex (r = 0.177, *P* = 0.016) or age (r = 0.161, *P* = 0.029). In addition, there were no correlations between levels of serum leptin and infarct volume (*P* = 0.090), or stroke subtypes (*P* = 0.258).

**Figure 2:**
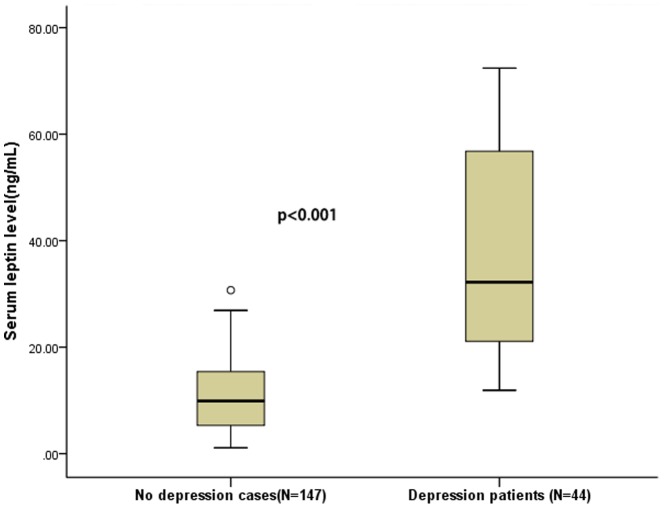
Serum leptin levels in acute ischemic stroke patients with depression and no depression. Mann–Whitney U-test. All data are medians and in-terquartile ranges (IQR). Significantly higher in stroke patients with depression as compared to no depression (P = 0.000).

There was a significant difference in median serum leptin levels between PSD patients and no depression cases (32.2 [IQR, 20.8–57.7] v. 9.9 [IQR, 4.6–13.1]ng/ml, respectively; *P*<0.001[z = 8.634]; Figure 2). Others variables relating to the presence of major depression at 3 month were: age (71.2 v.64.9, *P* = 0.009[t = 2.635]), sex (female 59.1% v. 39.5%, *P = 0.021*[t = 5.299]), BMI (26.8 v. 23.9, *P = 0.001*[t = 3.464]), widowhood (40.9% v.18.4%, *P* = 0.002[t = 9.555]) and living with offspring (36.4% v.13.6%, *P* = 0.001[t = 11.467]). Patients with major depression at 3 months showed higher serum levels of Hs-CRP and homocysteine (*P* = 0.003[z = 2.973] and *P* = 0.023[z = 2.255]; respectively). However, none of those two markers was associated with an increased risk of PSD, after adjustment for possible confounders (*P* = 0.341 and *P* = 0.526 respectively). See the table 2.

**Table 2:** Adjusted OR of depression for leptin levels at admission in the stroke patients

Parameter	OR	95% CI	P
Age(increase per unit)	1.05	1.02–1.10	0.012
Males	0.84	0.72–1.06	0.023
Widowhood	1.55	1.21–2.06	0.009
Living with offspring	1.63	1.06–2.46	0.018
NIHSS on admission(increase per unit)	1.31	0.89–2.26	0.236
BMI(increase per unit)	1.18	1.07–1.31	0.002
Hs-CRP(increase per unit)	1.13	0.79–2.25	0.341
HCY(increase per unit)	1.16	0.76–2.42	0.526
Leptin levels at admission(increase per unit)	1.15	1.05–1.25	<0.001
Leptin levels at admission(≥20 ng/ml)	20.23	9.11–51.26	<0.001

OR, odds ratio; CI, confidence interval; NIHSS, National Institutes of Health Stroke Scale; BMI, body mass index; Hs-CRP, High-sensitivity C-reactive protein; HCY, homocysteine.

**Figure 3:**
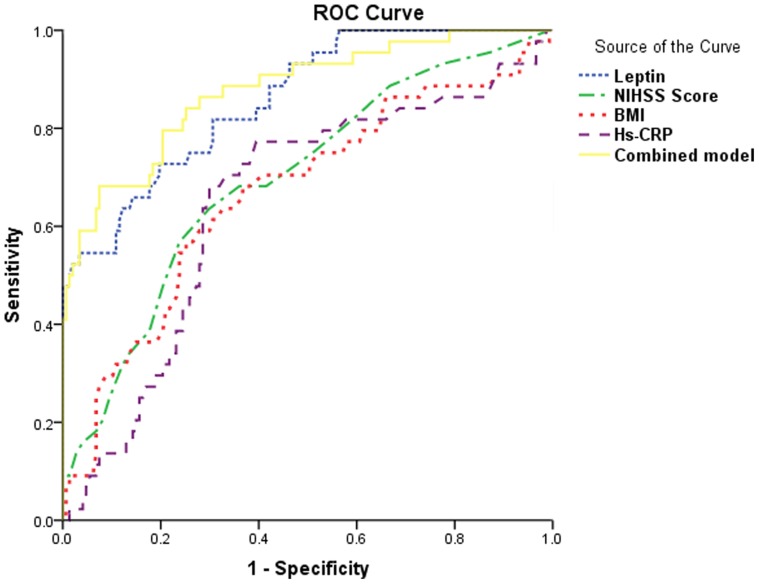
Receiver operator characteristic curve demonstrating sensitivity as a function of 1-specificity for predicting the depression within 3 month based on the combined model and the relative contribution of each marker alone (initial cohort). This combined model had an area under the receiver operator characteristic curve of 0.876(95%CI, 0.815–0.938) [0.697 (95%CI, 0.609–0.789) for NIHSS score; 0.667(95%CI, 0.571–0.763) for BMI; 0.650 (95%CI, 0.556–0.744) for Hs-CRP; 0.860 (95%CI, 0.799–0.924) for leptin].

Based on the ROC curve, the optimal cutoff value of serum leptin levels as an indicator for predicting of PSD was projected to be 20 ng/ml, which yielded a sensitivity of 81.8% and a specificity of 72.4%, with the area under the curve at 0.860(95%CI, 0.799—0.924). With an AUC of 0.860, leptin showed a significantly greater discriminatory ability as compared with Hs-CPR (AUC, 0.650; 95% CI, 0.556–0.744; *P* =  0.003), NIHSS score (AUC, 0.697; 95% CI, 0.609–0.789; *P* =  0.000) and BMI (AUC, 0.667; 95% CI, 0.571–0.763; *P* =  0.001). Interestingly, the combined model didn’t improve the leptin alone (AUC of the combined model, 0.876; 95% CI, 0.815–0.938; *P*<0.001). See the figure 3.

The serum levels of leptin determined at admission was the marker associated with the presence of major depression at 3 months (OR 1.21, 95% CI: 1.12–1.30; *P*<0.001). In the multivariate, leptin as a continuous variable was associated with an increased risk of PSD, after adjustment for possible confounders (OR 1.15, 95% CI: 1.10–1.25; *P*<0.001). See the table 2. Further, in our study, we found that an increased risk of PSD was associated with serum leptin levels ≥20 ng/ml (unadjusted OR 32.97, 95% CI: 13.35–81.44; *P*<0.001). This relationship was confirmed in the dose-response model. In multivariate analysis, there was an increased risk of PSD associated with serum leptin levels ≥20 ng/ml (OR 20.23, 95% CI:9.11–51.26; *P*<0.001) after adjusting for possible confounders. Interestingly, the association between PSD risk and serum leptin levels ≥20 ng/ml was more pronounced among men (adjusted OR 25.19, 95% CI: 7.12–63.12; *P* =  0.000) when compared with women (adjusted OR 14.22, 95% CI: 6.02–32.41; *P* = 0.000). See the table 2.

## Discussion

We found that patients with high leptin levels have a higher risk of developing major depression 3 months after stroke, even after adjustments for others risk factors. This correlation was more pronounced among men when compared with women. Consistent with our results, a recently published study including 104 patients with stroke showed that leptin was a powerful biological marker of risk of developing depression 1 month after stroke [29]. Similarly, Pasco et al [30] found that elevated serum leptin predicted subsequent development of a depressive disorder after adjusted BMI, medications or other lifestyle factors in female non-smokers.

Previous study found that the prevalence of PSD varies over time with an apparent peak 3–6 months after stroke and subsequent decline reaching about 50% of the initial rates at one year [6]. In our study, the prevalence of 3 months after stroke was 23.0%. Consistent with this, Jime′nez et al [29] reported that 22.1% of stroke patients were diagnosed as having major depression at 1 month after stroke.

In this study, we found that elevated serum leptin level was associated with PSD. In humans, hyperleptinemia has been associated with increased intima thickness and also seems to increase vascular calcification [31]. In addition, high leptin associates with left ventricular hypertrophy [32] in hypertensive subjects, independently of actual BP levels. Hyperleptinemia also associates with fibrinolytic abnormalities [33].

Interestingly, previous studies in humans have had different results, and both increased [34]–[35] and decreased [36]–[37]. Leptin levels in depressed patients were found, while no association was reported by Deuschle et al [38]. Furthermore, Milaneschi et al [39] reported that low leptin signaling rather than low leptin concentration is a risk factor for depression and the relationship between leptin blood concentrations and depressive symptoms was modified by abdominal adiposity.

Leptin may influence depressive symptoms via different biological mechanisms. Firstly, leptin induces neurogenesis and angiogenesis after stroke and leads to increased leptin receptor and pAMPK concentrations. Leptin receptors are expressed in the hippocampus and amygdala, suggesting a potential neuroactive function [40]. Leptin receptors are also present on endothelial cells may play role in atherogenesis and impairment of vascular function [17]. In vascular smooth muscle cells, leptin stimulates vascular smooth muscle proliferation and migration [41]. Secondly, leptin has also been shown to affect hippocampal and cortical structure through its actions on neurogenesis, axon growth, synaptogensis and dendritic morphology regulation [42]. Thirdly, elevated serum leptin in depression might in turn further promote corticotropin-releasing hormone (CRH) release, as shown in animals and, hence, contribute to HPA system hyperactivity seen in depression [43]. Fourthly, interestingly, it has been shown that leptin may partially mediate the pathway from expanded adipose tissue to pro-inflammatory biomarkers increase [39]. In animal models it has been shown that C-reactive protein, the acute phase protein produced by hepatocytes in response to pro-inflammatory cytokines stimulation, may directly bind to leptin and attenuate its physiological functions [44]. Furthermore, Penninx et al [45] indicated that depression was associated with obesity-related metabolic alterations, and leptin has been proposed to play a role in this process [46]. In this study, we also found a significantly positive correlation between serum levels of leptin and BMI (P = 0.001). In addition, obesity caused by leptin may be associated with other lifestyle factors considered bio-behavioral risk factors for depression, and obesity may increase psychological distress and depressive symptoms [39].

This study has a number of limitations. The major limitation of our study was that we were not able to examine the risk factors for depressive episodes such as, lack of social support, poverty, family violence, and increased life stress. Secondly, insulin was not measured in this study, while Söderberg et al [47] found a close association between circulating leptin and insulin levels. Thirdly, the serum level of leptin was tested only one time at admission, further studies are needed to assess how leptin levels change across time after stroke and whether levels drawn at later points provide improved prognostic information. Lastly, the study subjects were few (n = 191) and came from only one clinic. Therefore, our findings may not be generalizable to other Chinese stroke patients. Further research is needed.

In conclusion, in spite of these limitations, our findings of this study remain important and demonstrate a strong relationship between leptin serum levels at admission and the development of PSD within the 3 months in Chinese population. Further studies are advocated to confirm this association, which may provide new proposal for the treatment of PSD.
